# Reducing GABA_A_-mediated inhibition improves forelimb motor function after focal cortical stroke in mice

**DOI:** 10.1038/srep37823

**Published:** 2016-11-29

**Authors:** Claudia Alia, Cristina Spalletti, Stefano Lai, Alessandro Panarese, Silvestro Micera, Matteo Caleo

**Affiliations:** 1 Scuola Normale Superiore, 56126, Pisa, Italy; 2 CNR Neuroscience Institute, 56124, Pisa, Italy; 3 The BioRobotics Institute Scuola Superiore Sant’Anna, 56025, Pontedera (PI), Italy; 4 Ecole Polytechnique Federale de Lausanne (EPFL), Bertarelli Foundation Chair in Translational NeuroEngineering Laboratory, Center for Neuroprosthetics and Institute of Bioengineering, CH-1015 Lausanne, Switzerland

## Abstract

A deeper understanding of post-stroke plasticity is critical to devise more effective pharmacological and rehabilitative treatments. The GABAergic system is one of the key modulators of neuronal plasticity, and plays an important role in the control of “critical periods” during brain development. Here, we report a key role for GABAergic inhibition in functional restoration following ischemia in the adult mouse forelimb motor cortex. After stroke, the majority of cortical sites in peri-infarct areas evoked simultaneous movements of forelimb, hindlimb and tail, consistent with a loss of inhibitory signalling. Accordingly, we found a delayed decrease in several GABAergic markers that accompanied cortical reorganization. To test whether reductions in GABAergic signalling were causally involved in motor improvements, we treated animals during an early post-stroke period with a benzodiazepine inverse agonist, which impairs GABA_A_ receptor function. We found that hampering GABA_A_ signalling led to significant restoration of function in general motor tests (i.e., gridwalk and pellet reaching tasks), with no significant impact on the kinematics of reaching movements. Improvements were persistent as they remained detectable about three weeks after treatment. These data demonstrate a key role for GABAergic inhibition in limiting motor improvements after cortical stroke.

Recovery of arm function is a high priority for individuals with motor cortex injuries. Following cortical stroke, some spontaneous motor improvements occur within the first months after the insult, but the underlying mechanisms are only partly understood^1^,^2^,^3^,^4^.

Rodent models can provide detailed insights into the mechanisms responsible for spontaneous and rehabilitation-induced motor improvements. A variety of motor tests have been developed for these species^5^,^6^,^7^, and, in particular, skilled reaching in rodents shares many similarities with the homologous behavior in humans^8^.

Following an ischemic lesion, a substantial reorganization of both neural and glial/endothelial cells takes place in the injured area^4^,^9^. In animal models, reorganization of motor maps has been observed^10^,^11^,^12^ showing a post-stroke shrinkage of the paretic limb map, prevented by an early rehabilitation, which is also effective at improving motor function^13^,^14^,^15^.

Little is known about the cellular mechanisms that lead to these network reorganizations and recovery of the lost motor function. The GABAergic system and the extracellular matrix could have an important role in controlling plastic phenomena^16^. For example, Perineuronal Nets (PNNs), preferentially surrounding the soma of fast-spiking parvalbumin-positive interneurons^17^, and the GABAergic system have been extensively investigated during the maturation and plasticity of the visual system^18^,^19^,^20^. Moreover, following CNS injury, the degradation of PNNs promotes sensory-motor recovery^21^,^22^,^23^. Increasing GABAergic signalling after stroke is not effective at improving motor performances^16^, but rather produces a worsening of recovered function in stroke patients^24^. Conversely, a reduced GABAergic inhibition is associated with functional recovery^25^,^26^,^27^.

Ionotropic GABA_A_ receptors mainly mediate the inhibitory effect of GABA in the brain^28^,^29^. GABA_A_ receptors are composed of different subunits and the resulting molecular assembly determines the localization and biological action of the receptor. A general downregulation of GABA_A_ receptors has been found in peri-infarct areas following photothrombosis-induced lesions in rats and mice^30^,^31^,^32^. Moreover, an experimental reduction of post-stroke, excessive tonic GABA signalling produces significant post-stroke improvements of forelimb function^5^,^33^.

Here we have investigated how the restoration of lost motor function in mice is related to modulation of inhibitory GABAergic signalling following a targeted photothrombotic lesion to the forelimb primary motor area (Caudal Forelimb Area, CFA). We found that network reorganization after stroke was associated with a delayed reduction of “plasticity brakes” including PNNs and GABAergic markers. Thus, we targeted GABAergic signalling in an early post-stroke period, using methyl-6,7-dimethoxy-4-ethyl-beta-carboline-3-carboxylate (DMCM), an inverse benzodiazepine agonist that inhibits GABA currents^34^ by binding to the α subunits of the GABA_A_ receptor (affinity: α1 > α2 = α3 > α5)^35^,^36^,^37^. Specifically, DMCM acts by decreasing the affinity of the receptors for released GABA^38^ thus allowing the modulation of endogenous intracortical inhibition during post-stroke plasticity. Using sensitive behavioural readouts such as the gridwalk and pellet reaching tasks, we demonstrate that DMCM treatment caused significant and long-lasting motor improvements, with no significant impact on the kinematics of reaching movements.

## Results

### Deficits in forelimb function following stroke

We induced a focal injury in the caudal forelimb area (CFA) of the mouse motor cortex using Rose Bengal-mediated photothrombosis^39^. The ischemic lesion was confined to the targeted cortical area of the illuminated hemisphere. The histopathological analysis was conducted 30 days after the ischemic injury and showed that the lesion volume was 0.93 ± 0.21 mm^3^. Our typical lesion remained confined into the CFA and did not involve the Rostral Forelimb Area (between +1.5 and +2.25 mm anterior to bregma suture) and the hindlimb representation (between −0.75 and −1.25 mm posterior to bregma) according to average maps published in the literature^12^ (see also below). Coronal section of the ischemic brains demonstrated a complete loss of neurons in the core of the infarct, and well-defined boundaries that separate the lesion from the healthy perilesional tissue (Supplementary Fig. S1a).

To investigate the time-course of functional motor deficits, animals were tested on two motor tasks (gridwalk, Schallert cylinder) before the injury (baseline) and up to 30 days after the ischemic lesion (once a week starting from day 2). Supplementary Fig. S1b reports the percentage of foot faults done with the contralesional forelimb in the gridwalk test. After the ischemic injury, stroke mice (n = 11) showed a significant and stable increase in forelimb foot faults as compared to sham-treated animals (n = 9). Conversely no changes in foot faults were observed for the contralesional hindlimb, confirming the anatomical specificity of the stroke location (Supplementary Fig. S1c). The Asymmetry Index evaluated with the Schallert cylinder test showed increased reliance on the ipsilesional forelimb during exploration of the vertical walls in ischemic mice throughout the testing period (Supplementary Fig. S1d). Proficiency in other motor tests appears to improve spontaneously after injury, especially when end-point measures are used. Indeed, it has been clearly shown that sensory-motor performances (including reaching success in the pellet grasping task) improve already after 2 weeks^39^,^40^,^41^,^42^,^43^.

### Plastic modifications of motor maps after injury

Intracortical microstimulation (ICMS) was used to investigate the spontaneous remapping of motor areas, 30 days after the ischemic lesion. Specifically, we measured the extension and selectivity of the cortical representation of the affected forelimb in the intact areas surrounding the infarct.

**Figure 1:**
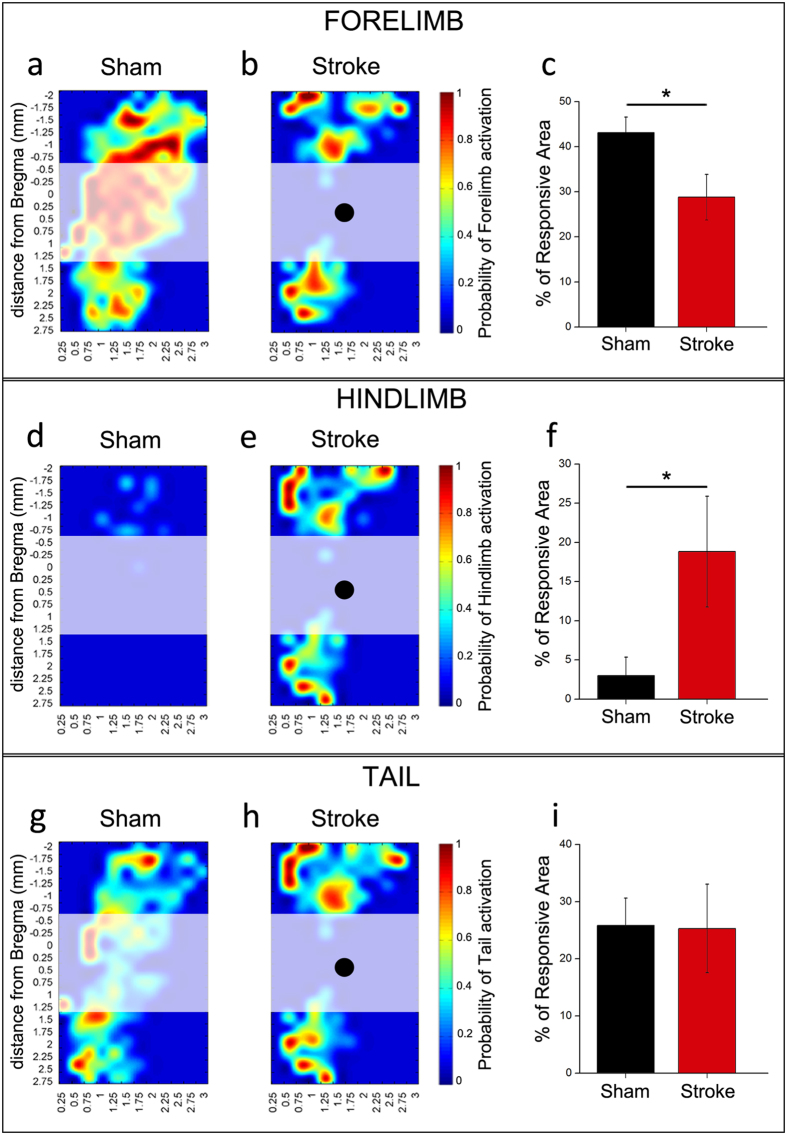
Plasticity of motor maps after stroke. Average motor maps of sham (n = 9) and ischemic animals (n = 6) obtained by the intracortical microstimulation (ICMS) technique following a grid of stimulation site of 250 μm (see coordinates from bregma and Fig. 2a). For each pixel in the maps, a color code indicates the probability (high – red; low – blue) to elicit movement of a body part (contralateral forelimb in (**a,b)**; contralateral hindlimb in (**d,e)**; tail in (**g,h**) after stimulation with a train of cathodal pulses (current, 30 μA). The quantitative analysis was performed in healthy regions rostral and caudal to the stroke, excluding the infarct zone, which is indicated by the shaded areas (black dots indicate the center of the lesion). (**c,f,i**) Quantification of the percentage of perilesional motor areas eliciting forelimb (**c**), hindlimb (**f**) and tail responses (**i**) in sham and stroke animals. After stroke, there is a significant decrease in the cortical area that evokes forelimb responses (t-test, p < 0.05), with a corresponding expansion of hindlimb motor maps (t-test, p < 0.05). Tail representations are not altered by the infarct (t-test, p = 0.551). Data are mean ± SE.

Forelimb motor maps from sham (n = 9) and stroke (n = 6) animals are compared in Fig. 1a–c. In control animals, the CFA is well defined and extends from approximately −1.0 mm posterior up to 1.25 mm anterior to the bregma suture, consistent with previous reports^44^. A second, smaller forelimb representation (Rostral Forelimb Area, RFA) is centred at about 2.5 mm anterior to bregma (Fig. 1a). This area displays a slightly higher current threshold for evoking limb movements as compared to the CFA (RFA, 36.5 ± 10.9 μA; CFA, 28.9 ± 5.7 μA; t-test, p = 0.08). After injury, the area corresponding to the infarct (shaded region in Fig. 1a,b) was no longer effective in triggering forelimb movements. Areas anterior and posterior to the lesion continued to evoke a forelimb response after stroke (Fig. 1b). However, the quantitative analysis, performed only in the healthy tissue (included between −0.75 mm and −2 mm and between +1.5 mm and +2.75 mm anteroposterior to bregma) indicated a significant shrinkage of the forelimb area in perilesional cortex of ischemic animals as compared to the corresponding regions in controls. In fact, the percentage of responding area in peri-infarct cortex indicated a reduced representation of the contralesional forelimb in ischemic mice (t-test, p = 0.03) (Fig. 1c). The effect was evident and significant across a range of stimulation intensities (20–40 μA; data not shown). It is also worth noting that the current threshold required for eliciting a forelimb movement in perilesional areas significantly increased after stroke (Supplementary Fig. S2a; t-test, p = 0.003). In normal animals, we also observed ipsilateral forelimb movements (percentage of responsive area was 26.9 ± 4.1). After stroke, there are not significant changes of the extension of the ipsilateral forelimb area in the spared motor cortex anterior and posterior to the lesion (23.0 ± 7.5; t-test, p = 0.63).

During ICMS, we also examined movements of other body parts such as the hindlimbs and tail. In normal animals, the contralateral hindlimb and tail representations are smaller than the forelimb and occupy the caudal extent of primary motor cortex^12^. Interestingly, we found that the proportion of sites mapping the contralesional hindlimb increased in the perilesional cortex of ischemic mice, thus invading forelimb representation (t-test, p = 0.04) (Fig. 1d–f). There was also a non-significant trend towards a post-stroke reduction in the current threshold required for eliciting hindlimb movements in forelimb area (Supplementary Fig. S2b; t-test, p = 0.148). Expansion of the hindlimb map was evident and significant at various stimulation currents (20–60 μA). In contrast, the overall dimensions of the tail map did not change significantly in stroke vs. control mice (t-test, p = 0.551) (Fig. 1g–i). During stimulation, we observed very sporadic ipsilateral hindlimb movements (percentage of responding area 2.5 ± 2.4). After stroke the area eliciting a ipsilateral hindlimb movement was increased (11.2 ± 4.9) but this trend did not reach statistical significance (t-test, p = 0.1).

**Figure 2:**
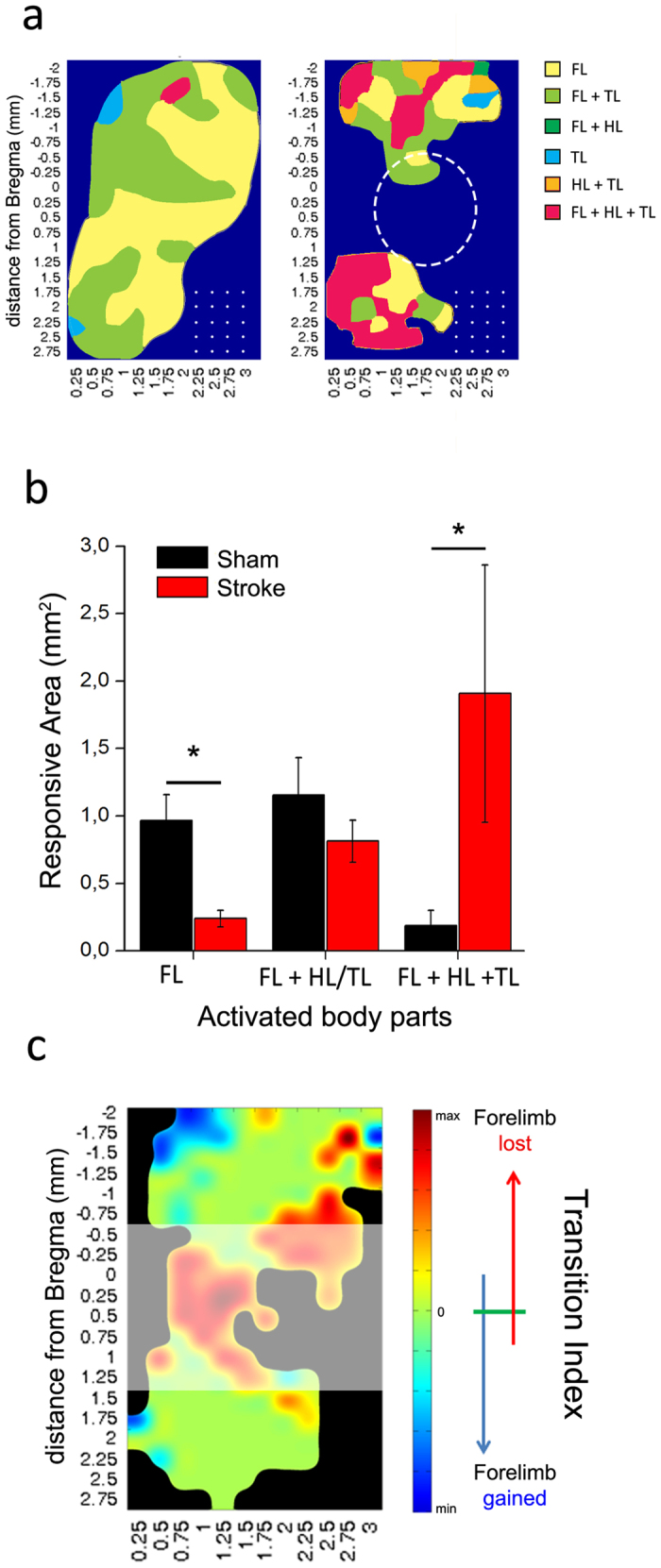
Loss of movement selectivity following a cortical infarct. (**a**) Scheme of the average map showing major changes in forelimb (FL), hindlimb (HL) and tail (TL) representation after stroke. Colors shows the preferential activation of different regions, showing the specificity of each zone (single vs multiple movements). Dotted circle indicates the average position of our ischemic lesion and white dots represent the grid of stimulation used in ICMS experiments. (**b**) Surface area eliciting movement of forelimb (FL) alone, forelimb + hindlimb (HL) or tail (TL), or the three body parts together at a stimulation current of 30 μA in sham (black, n = 9) and ischemic animals (red, n = 6). The quantitative analysis was performed in regions rostral and caudal to the infarct (see Fig. 1). Following stroke, very few sites retain their selectivity for forelimb movements, while most cortical locations elicit simultaneous movement of forelimb, hindlimb and tail (t-test, p < 0.05). Data are mean ± SE. (**c**) Map of the Transition Index (TI; see Methods) showing for each pixel the tendency to gain (blue) or lose (red) forelimb movement after stroke in our experimental sample. The colorimetric index is defined within a range of 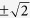 (see Methods).

Overall, these data indicate a significant reduction of the size of forelimb representations in the intact perilesional tissue, with corresponding expansion and invasion of hindlimb maps. We next investigated whether the functional selectivity of the perilesional tissue was affected in stroke vs. control animals. Selectivity was quantified by counting the proportion of cortical sites evoking movement of 1, 2 or 3 parts of the body (i.e., contralesional forelimb, contralesional hindlimb and tail) using a 30 μA stimulation current. We found that the majority of sites in normal animals evoke the movement of either 1 or 2 body parts, whereas very few sites evoke simultaneous movements of forelimb, hindlimb, and tail (Fig. 2a,b). The picture was radically different in ischemic animals, where very few sites were specific for the forelimb (t-test, p = 0.011), and the majority of cortical locations drove movements of 2 or 3 body parts, even at low current intensities (t-test, p = 0.046; Fig. 2b).

We also investigated whether specific locations in the perilesional cortex consistently lose or gain forelimb preference following a cortical infarct. To this aim, we computed a “Transition Index” (TI) (see Methods) that indicates with a colorimetric scale if each cortical site gains (blue) or loses (red) forelimb movements after stroke (Fig. 2c). Overall, a robust loss of forelimb sites was evident both anterior and posterior to the infarct, with the exception of a postero-medial area (blue) that appears to gain forelimb responses in stroke vs. sham mice (Fig. 2c).

### Downregulation of GABAergic markers following stroke

We next used immunostaining to evaluate expression of markers of plasticity^18^,^45^ in perilesional cortical areas. We performed staining for perineuronal nets (PNNs), somatostatin and parvalbumin-positive (SOM- and PV-positive) inhibitory interneurons, and glutamatergic and GABAergic terminals. Three groups of animals were examined: a sham group with no injury (n = 6), and ischemic animals at either 7 (n = 8) or 30 days post-lesion (n = 8). The analysis was performed in cortical areas adjacent to the infarct as illustrated in Supplementary Fig. S3. To ensure consistency among animals and lesions, we sampled well defined areas in the perilesional region. Specifically, the density of PNNs, PV- and SOM-positive cells was determined in cortical columns (200 μm wide) medial and lateral to the lesion, while glutamatergic and GABAergic synaptic terminals were sampled in counting boxes located in both superficial and deep layers of peri-infarct cortex (Supplementary Fig. S3). Homotopic regions of the contralesional hemisphere were examined as well, but we did not detect any significant changes in ischemic animals with respect to sham controls (data not shown).

**Figure 3:**
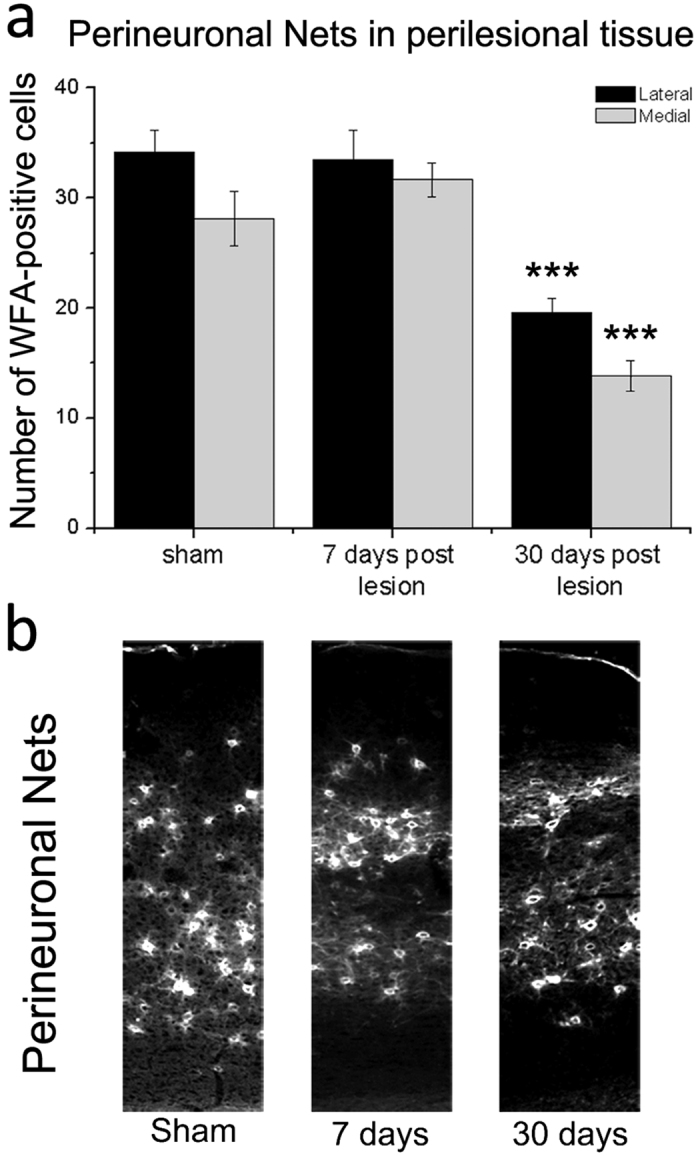
Reduced density of PNNs in peri-infarct areas. (**a**) Number of cells surrounded by PNNs in 200 μm wide cortical columns, lateral (black bars) and medial (grey bars) to the ischemic lesion, 7 (n = 5) and 30 days post injury (n = 8). Similar cortical regions were also sampled in controls (n = 8). PNN density is significantly reduced at 30 but not 7 days after stroke (one Way ANOVA, post hoc Holm-Sidak test, ***p < 0.001). Data are mean ± SE. (**b**) Representative images acquired from coronal sections of the motor cortex from sham and stroke mice at 7 and 30 days. Column width = 200 μm.

**Figure 4:**
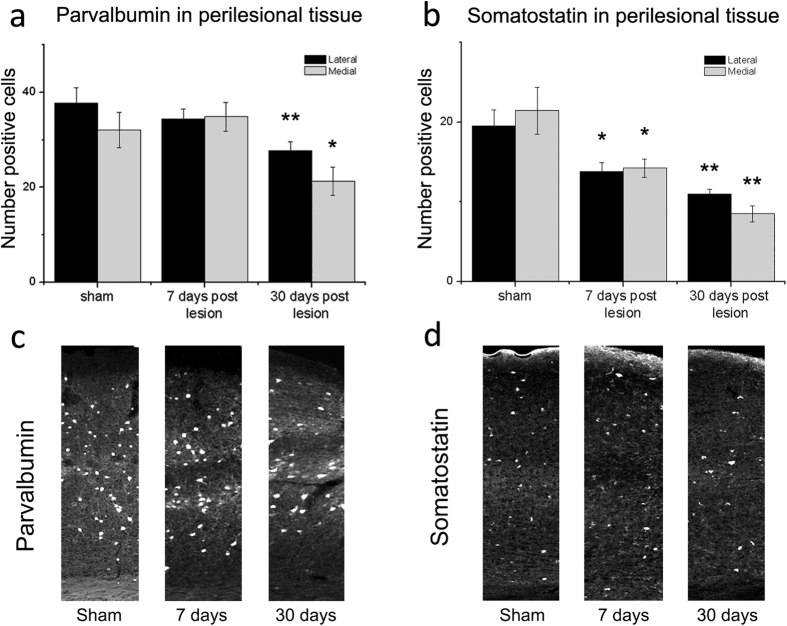
Reduced density of GABAergic interneurons in peri-infarct areas. (**a,b**) Number of PV-positive (**a**) and SOM-positive cells (**b**) in 200 μm wide cortical columns, lateral (black bars) and medial (grey bars) to the ischemic lesion, 7 (PV n = 7; SOM n = 6) and 30 days post injury (PV n = 8; SOM n = 4). Compared to controls (PV n = 7; SOM n = 6), density of PV-+ neurons is significantly dampened at 30 days (one Way ANOVA, post hoc Holm-Sidak test, p < 0.05). SOM-+ cells are significantly reduced at 7 days and decrease further at 30 days. Data are mean ± SE. *p < 0.05; **p < 0.01. (**c,d**) Representative low magnification images of PV (**c**) and SOM immunostaining (**d**) in the motor cortex. Images were taken from sham animals and from the perilesional areas of mice at 7 and 30 days post-infarct. Column width = 200 μm.

The density of PNNs was assessed by staining cortical sections with Wisteria Floribunda Agglutinin (WFA). PNNs enwrap specific sub-populations of neurons and are known to play a key role in limiting adult plasticity^18^,^22^,^23^. We found that the density of PNNs remained unaltered at day 7 but consistently decreased 30 days after stroke in cortical columns medial and lateral to the infarct (one way ANOVA followed by Holm-Sidak test, medial and lateral, p < 0.001; Fig. 3). Since PNNs mainly surround PV-positive cells (corresponding mainly to fast-spiking inhibitory neurons), we then measured the density of this neural population. PV-positive cells showed the same trend as PNNs, and were significantly downregulated 30 but not 7 days after stroke (one way ANOVA, followed by Holm-Sidak medial p = 0.027; lateral p = 0.009; Fig. 4a). Another important class of inhibitory interneurons, namely SOM-positive cells, also showed a consistent downregulation following injury. In this case, a statistically significant decrease in cell density was already noted at day 7 (one way ANOVA, followed by Holm-Sidak test, medial p = 0.023; lateral p = 0.017), and further progressed at day 30 (medial p = 0.001, lateral p = 0.003; Fig. 4b).

**Figure 5:**
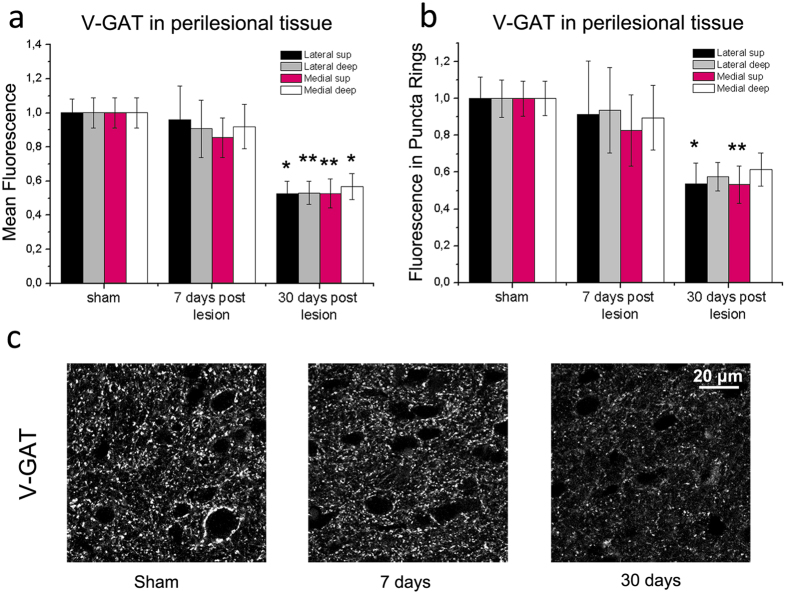
Late reduction of V-GAT-positive inhibitory terminals in perilesional cortex. (**a,b**) Mean fluorescence intensity of inhibitory, V-GAT-positive profiles in the neuropil (**a**) and in puncta rings surrounding the soma of pyramidal neurons (**b**). Measures were taken in superficial (sup) and deep layers, medial and lateral to the infarct. A consistent reduction in fluorescence is observed 30 but not 7 days after injury as compared to sham controls (one way ANOVA followed by Holm-Sidak test, p < 0.05). Data are mean ± SE. *p < 0.05; **p < 0.01. (**c**) Representative V-GAT immunoreactivity in the motor cortex of sham animals and in perilesional tissues (7 and 30 days after the infarct). Note decreased staining at 30 days. Number of animals is as follows: control n = 5, stroke 7 days n = 5, stroke 30 days n = 4. Scale bar = 20 μm.

To study more precisely the excitation/inhibition ratio in perilesional cortex, we stained sections for vesicular transporter markers that label inhibitory and excitatory terminals impinging onto cortical neurons^46^,^47^. Quantitative analysis of the immunostaining for the Vesicular GABA Transporter (V-GAT) indicated that the overall fluorescence of inhibitory terminals was unaffected at day 7, but robustly declined at day 30. This reduction was noted in superficial and deep layers on both sides of the infarct (one way ANOVA, followed by Holm-Sidak test, p < 0.01 for all comparisons; Fig. 5a,c). We also quantified perisomatic staining by measuring the mean V-GAT fluorescence in “puncta rings” surrounding cortical neurons^18^,^48^. We detected a decrease of fluorescence 30 days after stroke that was significant in superficial layers (one way ANOVA, followed by Holm-Sidak test, medial p < 0.01; lateral p < 0.05; Fig. 5b,c).

**Figure 6:**
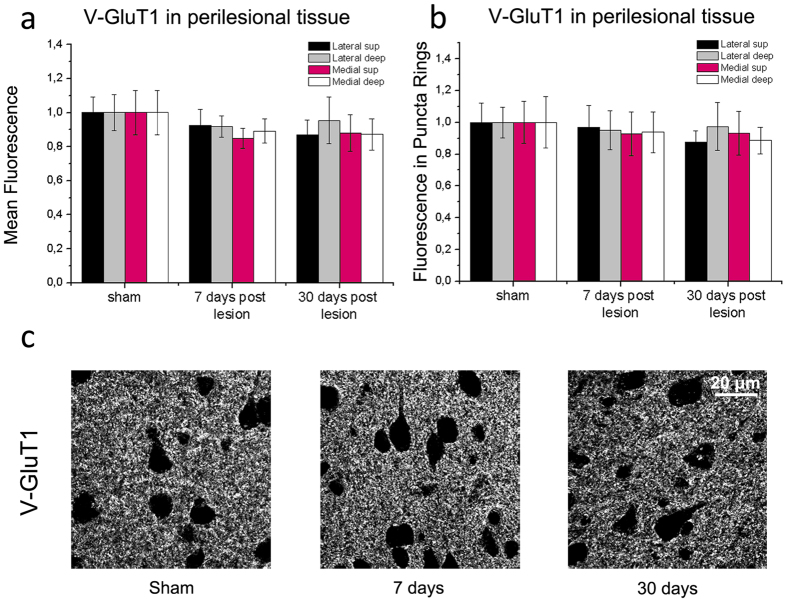
Quantitative analysis of V-GluT1 immunostaining. (**a,b**) Mean fluorescence intensity of excitatory, V-GluT1-positive presynaptic structures in the neuropil (**a**) and in puncta rings surrounding the soma of pyramidal neurons (**b**). Measures were taken in superficial (sup) and deep layers, medial and lateral to the infarct. There is no significant variation in staining in stroke animals as compared to sham controls (one Way ANOVA, followed by Holm-Sidak test, p > 0.52). Data are mean ± SE. (**c**) Representative V-GluT1 staining in cortical sections from sham controls and stroke mice, 7 and 30 days following injury. Number of animals is as follows: control n = 6, stroke 7days n = 6, stroke 30 days n = 5. Scale bar = 20 μm.

To evaluate excitatory terminals, we quantified immunostaining for the Vesicular Glutamate Transporter 1 (V-GluT1) in lateral and medial perilesional cortex. We found no significant changes in the mean V-GluT1 fluorescence in the neuropil either at 7 or 30 days after stroke (one way ANOVA, p > 0.52 for all peri-infarct regions; Fig. 6a–c). Similarly, the intensity of perisomatic excitatory boutons showed no significant variation after stroke (p > 0.63; Fig. 6b,c).

Overall, these data show a consistent downregulation of PNNs and several inhibitory markers in the peri-infarct cortex 30 days after stroke. Conversely, we found no detectable variation in V-GluT1 expression, indicating an overall shift of the excitation/inhibition ratio in favour of excitation. Increases in the excitation/inhibition balance are typically associated with an enhanced potential for plasticity in the adult cortex^20^,^49^,^50^, indicating their possible involvement in the spontaneous functional improvements occurring within the first weeks after stroke^39^,^40^,^41^,^42^,^43^.

### Interference with GABA_A_ signalling promotes functional restoration

The post-stroke reduction of GABAergic markers prompted us to investigate the role of GABA signalling in motor improvements. We decided to hamper GABA_A_ mediated neurotransmission during an early phase post-lesion (days 3–8) via systemic delivery of the benzodiazepine inverse agonist DMCM. We initially established doses of DMCM (1.5 mg/kg) that were well below those required to elicit epileptic seizures^51^,^52^,^53^, while at the same time impacting on GABA-mediated transmission as judged by a reduced path length in the open field^52^ (Supplementary Fig. S4).

**Figure 7:**
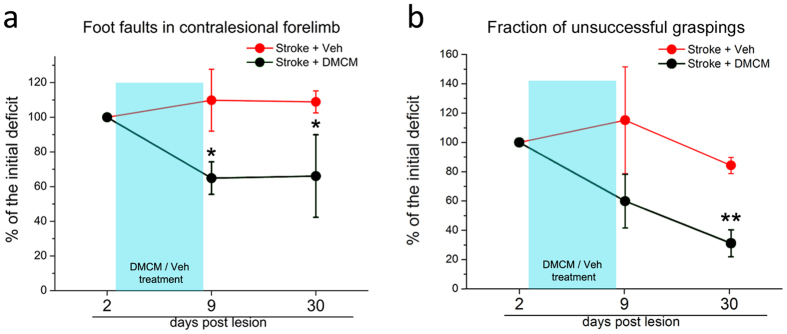
Long-lasting improvements in general motor outcome following DMCM treatment in stroke mice. Data are shown as percentage of the initial deficit, i.e. the difference in the fraction of foot faults/incorrect graspings between day 2 and baseline. Then, performances at day 9 and 30 have been normalized as a percentage of the initial deficit. Statistical analysis was performed on raw data and differences refer to day 2. (**a**) The number of foot faults in the gridwalk task decreases immediately after DMCM treatment and persists up to 30 days (two way RM ANOVA followed by Tukey test, p < 0.05; n = 10). Conversely, no significant improvements are detected in controls (p > 0.87; n = 3). (**b**) Performance in the single-pellet retrieval task. The fraction of incorrect graspings decreases after DMCM treatment, which rescues approximately 70% of the initial deficit 30 days post lesion (two way RM ANOVA, followed by Tukey test, p < 0.01). Data are mean ± SE.*p < 0.05; **p < 0.01.

Mice were treated with either DMCM (n = 10) or vehicle (n = 3) starting from day 3 after stroke and following assessment of the initial deficit in the gridwalk task (Fig. 7a). Injections were made daily until day 8 and motor function was again assessed at day 9 (i.e. immediately after treatment) and 30 (to probe persistence of the therapeutic effect). As expected, vehicle-treated mice showed no improvements in motor function. Importantly, a reduction in the number of foot faults was evident for DMCM-treated animals at day 9 and persisted at day 30 (two way RM ANOVA followed by Tukey test, day 9, p = 0.039; day 30, p = 0.038; Fig. 7a). We also assessed the impact of DMCM treatment on skilled reaching^39^ (DMCM n = 3; veh n = 3). The percentage of incorrect movements decreased at day 9 showing a trend for improvement in DMCM animals. This effect of DMCM became significant at day 30 (two way RM ANOVA followed by Tukey test, p = 0.007; Fig. 7b). Thus, performance in two motor tasks was consistently enhanced in the DMCM animals well beyond the completion of the treatment. This effect was not due to a difference in the initial deficit, since DMCM and vehicle groups had a comparable performance 2 days post lesion (two way RM ANOVA p > 0.095). We also quantified the number of total attempts made during the tests (i.e. reaching attempts in the skilled reaching test and steps performed in the gridwalk test) and we found no significant differences with respect to vehicle controls at 30 days post lesion (total steps made in the gridwalk test: DMCM 619.7 ± 96.46; Veh 635 ± 19.22; t-test p = 0.932; total attempts in the skilled reaching test: DMCM 63.6 ± 6; Veh 56 ± 7; t-test p = 0.454).

**Figure 8:**
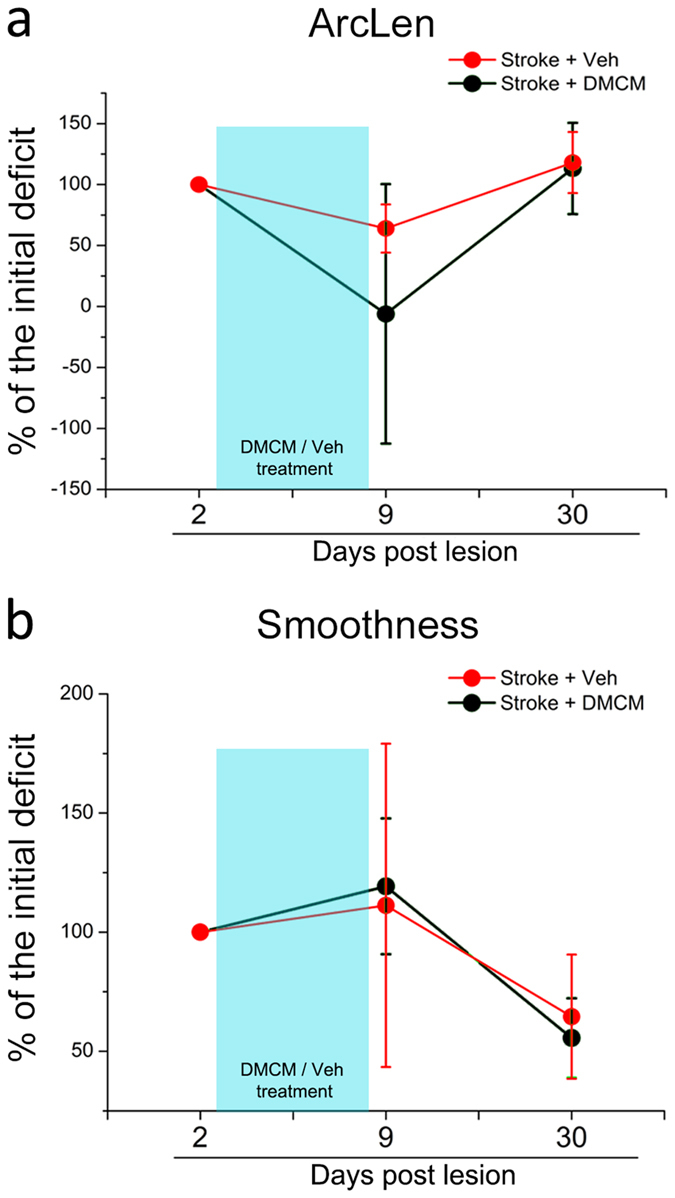
Kinematic analysis of pellet reaching indicates compensatory adjustments in DMCM-treated stroke mice. Data are expressed as percentage of the initial deficit. Post-stroke variation in the length of the whole trajectory (ArcLen; **a**) and in the smoothness of movement (**b**) during successful pellet retrievals in DMCM- (n = 3) and vehicle-treated stroke mice (n = 3). No significant differences are detected between the two groups (two way RM ANOVA, p > 0.24).

In the subset of the animals employed for the pellet grasping task, we performed a kinematic analysis of reaching to address the issue of “true” recovery vs. compensation^39^. We found that DMCM treatment did not significantly improve trajectory kinematics, as they remained longer (ArcLen, Two way RM ANOVA, DMCM vs. vehicle, p = 0.244; Fig. 8a) and less smooth (i.e., with an increased number of trajectory adjustments; Two way RM ANOVA, DMCM vs. vehicle, p = 0.330; Fig. 8b) as compared to baseline, pre-stroke values. Overall, these data indicate that hampering GABA_A_ signalling after stroke produces significant and long-lasting improvements in motor function, and compensatory adjustments appear to be required for task accomplishment.

## Discussion

Understanding cellular mechanisms governing post-stroke cortical plasticity is critical to develop novel and more effective pharmacological therapies to improve outcome in stroke survivors. The data reported in this manuscript indicate a substantial remodelling of GABAergic networks during post-stroke map reorganization of spared forelimb motor cortex in mice. Importantly, early experimental interference with GABA_A_ signalling triggered long-lasting restoration of forelimb function following a focal cortical infarct.

We focused our analysis on peri-infarct regions since evidence in patients and animal models indicate that spared areas surrounding the lesion significantly contribute to functional recovery^11^,^43^,^54^,^55^. In ICMS experiments, we observed a reduction in the number of cortical sites that elicited movements of the lesioned forelimb 30 days after stroke. This was accompanied by a corresponding invasion of the hindlimb motor map, that in healthy animals is centred posterior to the CFA^56^,^57^. Our results are consistent with previous reports indicating shrinkage of the cortical representation of affected forelimb in favour of other body parts^10^,^58^. These motor map reorganizations are reminiscent of activity-dependent competition in sensory cortices^18^,^59^,^60^.

The current threshold to evoke movements also changed after stroke. Overall, a higher current intensity was required to evoke a forelimb movement post-stroke, while hindlimb movements were elicited with a slightly reduced current. In keeping with our results, previous studies in humans and rodents have shown an inverse correlation between the current threshold and the size of the motor map^61^,^62^. Young and colleagues (2011) correlated GABAergic neurotransmission with cortical map properties, so that a shift in the balance between excitation and inhibition influenced both movement thresholds and map size. It has also been suggested that inhibitory neurotransmission is responsible for the stability and sharpening of motor and sensory maps^13^,^63^,^64^. Specifically, local reduction of intracortical inhibition allows the unmasking of subthreshold inputs and the consequent expansion of adjacent motor maps^65^. In this context, a reduction of GABAergic inhibition might allow plasticity of spared areas and pathways following stroke. Indeed, our neuroanatomical analyses revealed a significant reduction of two major populations of GABAergic cortical interneurons after the infarct. Somatostatin-containing interneurons represent a low-threshold inhibitory population involved in cortical plasticity^66^,^67^,^68^ whereas parvalbumin-positive fast-spiking interneurons play important roles in control of network firing due to their perisomatic synapses onto pyramidal cells^48^. It remains to be established whether loss of SOM and PV immunoreactivity is due to cell loss or down-regulation of the expression of these markers. However, reduction of GABAergic neurotransmission was confirmed by analysis of the number of V-GAT-positive inhibitory presynaptic terminals in the perilesional cortex, 30 but not 7 days after stroke. For a reliable quantification of immunofluorescence, we used a reference field for each analysed section (see methods) that reduces bias due to different quality of staining in individual samples. As compared to other more quantitative methods (such as Western blotting), this analysis has the advantage of ensuring greater spatial selectivity. A reduced inhibitory tone is in keeping with the observation of a loss of selectivity in the motor maps, with a dramatic enhancement in the number of sites evoking simultaneous movement of different body parts, and a corresponding reduction in the percentage of cortical area evoking specific and unique movements (see Fig. 2a,b).

Overall, our data point to a consistent downregulation of the GABAergic system that is initially detectable 7 days after stroke and becomes more widespread one month post-stroke, coupled with a reduction of PNNs. It has been shown that daily motor rehabilitation in stroke mice triggers an improvement of grasping abilities, which is associated with a reduction in parvalbumin, calretinin, and calbindin expression in the agranular cortex adjacent to the infarct. These exercise-dependent reductions in inhibition occur in the first week after stroke and are not evident without rehabilitation^69^. Overall, inhibitory networks appear to reorganize extensively in a quite large cortical area surrounding the infarct^70^, which may explain the changes in cortical maps seen by intracortical stimulation (present study) and optogenetics^11^.

It has also been shown that removing PNNs from the perilesional tissue can be beneficial for motor recovery after injury^22^,^23^. Thus, the release of specific “neuroplasticity brakes” (such as GABAergic neurotransmission and PNNs^18^,^45^) from the perilesional areas may trigger the reopening of a sensitive period for cortical remodelling that allows network reorganization and potentially some restoration of function. This interpretation fits well with the finding that, despite a global shrinkage of the affected forelimb representation, specific postero-medial sites of the motor cortex appear to gain forelimb responsiveness after stroke (blue “hot spots” in Fig. 2c) indicating a difference between the caudal and rostral regions of the motor cortex in post stroke motor recovery. This finding may indicate an initial attempt of the motor network to relocalize lost motor functions. Interestingly, Starkey *et al*.^43^ observed that in stroke rats with good functional outcome, neurons within the hindlimb motor cortex (posterior to the lesion site) acquire the ability of triggering forelimb movements, pointing to compensatory take-over by peri-lesional areas.

Several studies have reported a decreased intracortical inhibition in stroke patients^71^. After unilateral stroke, Rossini and colleagues found a reduction of intracortical inhibition in the affected hemisphere and normal inhibitory levels in the contralesional one^73^. Kim *et al*.^25^ reported reduced GABA_A_ receptor availability in the primary motor cortex of stroke patients and these changes in GABA receptor availability were significantly related to motor recovery^25^. In the present experiments, the downregulation of GABA markers at 30 days was accompanied by a trend for spontaneous improvement in the skilled reaching test (see in Fig. 7b). Of note, our animals were not tested daily, differently from previous reports where major, training-induced improvements in this task have been described^43^,^69^. Altogether, these data are consistent with the view that a reduction of the inhibitory tone generally facilitates the reinstatement of plasticity in the adult cortex^49^.

Based on these premises, we downregulated GABAergic signalling early after stroke in order to boost such spontaneous plastic phenomena. Increasing GABA signalling in post stroke patients and rodent models does not improve motor function^16^. Rather, administering a benzodiazepine after rehabilitation leads to an acute loss of the regained function^24^,^74^. Finally, lowering tonic GABAergic inhibition improves motor recovery after stroke^5^,^33^. Here, we targeted GABAergic signalling by using the benzodiazepine inverse agonist DMCM, which reduces inhibitory currents by binding predominantly to synaptic GABA_A_ receptor subunits (DMCM binding affinity α1 > α2 = α3 > α5^35^). DMCM has been previously used to modulate plasticity in the visual cortex^63^. Our results show a significant improvement in behavioural readouts immediately after the treatment, i.e. day 9 post-stroke, which was maintained in the follow-up period up to 30 days after stroke. Improvements were seen both in general motor coordination and in skilled forelimb use, as shown by the gridwalk and pellet grasping task, respectively. Improvements were not due to the anxiogenic properties of the drug^52^, as the motor performance was maintained or even enhanced at 30 days after termination of the treatment (see Fig. 7).

We chose to administer DMCM during an early post-stroke phase (day 3 - day 8) corresponding to the period of maximal susceptibility to therapeutic interventions^75^. Reducing GABA neurotransmission too early can have a detrimental effect on lesion size and motor outcome^5^. Indeed, the inhibitory system is believed to dampen excitotoxic phenomena in the acute phase, thus limiting the extent of damage. On the other hand, previous studies have shown that an early therapeutic intervention appears to be critical for the motor outcome^41^,^76^. These data suggest the existence of a “critical period” during which intervention strategies are more effective. These post-stroke events are reminiscent of the sensitive periods in developing sensory cortices, determined by an optimal level of GABA_A_ signalling^20^. In animal models, expression of GABA_A_ receptor subunits shows a complex layer- and time-specific regulation after stroke. GABA_A_ receptors generally decrease in perilesional cortical areas^5^,^32^,^70^,^77^,^78^,^79^. Conversely, one recent report^80^ has shown that α1 subunit expression is increased in layer 5 after stroke and its activation using zolpidem improves recovery. These findings are difficult to reconcile with the present report, where the antagonism of the GABA receptors (mainly α1) appears to promote recovery. The complex post-stroke changes in composition and localization of the GABA receptors make it diffcult to clarify the precise site of action of the GABA-acting drugs. In particular zolpidem, even if believed to work at the synapse, can act also at the extrasynaptic level^81^, adding complexity to the interpretation of its mechanism of action. In our experiments, reducing GABA_A_ signaling via DMCM results in a significant gain of forelimb function after stroke, possibly by adjusting the excitation/inhibition balance in specific cortical microcircuits^20^. Whether DMCM acts at the level of the perilesional cortex, contralesional hemisphere or subcortical structures remains to be addressed. Overall, downregulation of GABA_A_ signalling during an early post-stroke period appears to leave a persistent trace on post-stroke networks that translate into a long-lasting motor improvement.

Altogether, our data highlight a coordinated remodelling of GABAergic inhibitory networks that play an important role in post-stroke behavioural gains. Pharmacological therapies impacting on GABAergic signalling may accelerate and improve such spontaneous plastic adjustments resulting in significant, even if not complete, restoration of motor function. In fact, DMCM treatment was effective to improve general motor and skilled task but not the kinematics of reaching movements, possibly because of the lack of a specific rehabilitation that guides the re-establishment or pre-lesion motor patterns. It is possible that an appropriate combination of reduced GABAergic signalling and motor training leads to synergic effects resulting in a more complete post-stroke motor recovery^2^. In keeping with this idea recent studies have reported an additive or synergic effect of motor rehabilitation with plasticizing treatments, e.g. treatment with chondroitinase ABC was potentiated by task specific rehabilitation in spinalized rats^82^. In this context we recently introduced a robotic platform that allows animals to perform repeated controlled sessions of forelimb retraction and represents a promising tool for intensive and specific forepaw motor training^83^.

## Materials and Methods

### Experimental design

All procedures were performed in accordance with the EU Council Directive 2010/63/EU on the protection of animals used for scientific purposes, and were approved by the Italian Ministry of Health. A total of 60 C57BL6J mice were used (22–27 g, age 8–10 weeks).

As shown in Supplementary Fig. S5, a subset of these animals was used to assess behavioural, functional and histological changes after focal cortical stroke in CFA (n = 18), compared with control animals (n = 23). In order to investigate the time-course of functional motor deficits along the post-stroke acute-phase (i.e. up to 30 days after infarct), a baseline session for general motor tests (gridwalk and Schallert cylinder tests) was acquired before the photothrombotic (n = 11) or sham (n = 9) procedure and mice were retested in motor tests on days 2, 9, 16, 23, and 30 post lesion. In order to examine plasticity markers, a subgroup of stroke (n = 8) and sham (n = 8) animals underwent paraformaldehyde fixation at the end of the behavioural evaluation period (i.e. at 30 days post-stroke). Additional 6 stroke animals were examined at 7 days post-lesion. Moreover, we evaluated functional remapping of forelimb representation through Intracortical microstimulation (ICMS) experiments 30 days after injury in a total of 6 photothrombotic animals, compared with 9 control mice.

Based on the data obtained in the first part of this study (see Results), we planned a second experiment to evaluate if reductions in GABAergic signalling were causally involved in motor improvements. In order to establish a suitable dose of DMCM we performed the open field test in DMCM (n = 3) or vehicle (n = 3) mice.

To assess the impact of DMCM on post-stroke recovery, a second pool of 13 animals was tested in the gridwalk and Schallert cylinder tests to establish a pre-injury baseline performance. Among them, 6 mice had been also trained for two weeks in a custom-made skilled reaching apparatus^39^ until the performance showed a plateau, to collect at least three baseline sessions. All animals underwent cortical stroke and then were randomly assigned to the DMCM group (n = 10, 3 of which performed also the skilled reaching test) or vehicle group, i.e. saline solution, (n = 3). DMCM/vehicle was delivered from day 3 to day 8 post-lesion and motor tests were performed on days 2, 9 and 30.

### Photothrombotic lesion

The photothrombotic lesion was induced as previously described^39^. Briefly, after a midline scalp incision, Rose Bengal (0.2 ml of a 10 mg/ml solution in PBS; Sigma Aldrich) was injected intraperitoneally and the brain was illuminated through the intact skull for 15 min using a cold light source (ZEISS CL 6000) positioned 0.5 mm anterior and 1.75 mm lateral from Bregma (i.e. in correspondence with the caudal forelimb area; Tennant *et al*.^44^; Vallone *et al*.^84^). (see supplementary information).

### Motor tests

#### Gridwalk Test

Animals were allowed to walk freely for 5 minutes on an elevated grid (32 × 20 cm, with 11 × 11 mm-large openings) and the task was video-recorded. The video recordings were analyzed off-line by means of a custom-designed Graphical User Interface implemented in Matlab^39^, to assess correct steps and foot-faults, i.e. steps not providing body support, with the foot falling into grid hole. The percentage of foot faults for each limb was then calculated, as previously described^39^ by a blinded experimenter.

#### Schallert Cylinder Test

Animals were placed in a Plexiglas cylinder (8 cm diameter, 15 cm height) and recorded for 5 minutes by a video-camera placed below the cylinder. Videos were analyzed frame by frame and the spontaneous use of both forelimbs was assessed during exploration of the walls, by counting the number of contacts performed by the paws of the animal. The experimenter was blinded to the experimental group. For each wall exploration, the last paw that left and the first paw that contacted the wall or the ground were assessed. In order to quantify forelimb-use asymmetry displayed by the animal, an Asymmetry Index was computed, according to Lai *et al*.^39^.

#### Skilled Reaching Test and Kinematic Analysis

The percentage of correct movements and the kinematic analysis of the whole reaching movements were performed as previously described^39^. Briefly, animals (food deprived for 15 hours) were placed in a testing chamber with transparent walls and trained to perform a skilled reaching task with their preferred paw, which had to pass through a small frontal rectangular aperture (0.5 × 1.3 cm) to grasp and retrieve food pellets. The task was recorded by a high frame-rate video camera (Hero 3, GO-Pro, USA), placed on the side of the testing chamber thus allowing for a sagittal view of the animal. The shaping phase consisted in 2 days of habituation, in which a few pellets were placed inside the chamber. During the next phase, animals were trained daily until they reached 30 pellets in a single session (each lasting maximum 20 minutes). Animals were trained for a couple of weeks until they reached a plateau in the performance. The baseline value was obtained using the average of the last 3 sessions prior to the stroke. After the ischemic lesion animals were tested once a week without any additional training sessions.

The number of correct (i.e., a reach and grasp movements ending with pellet eating) and incorrect movements (i.e., when the mouse passed by the frontal window and reached the pellet but missed it or dropped the pellet after grasping it) were manually assessed. Then the percentage of incorrect grasping was calculated over the total attempts (i.e. total number of times that the paw crossed the frontal window.

Off-line reconstruction of paw trajectories was performed by a semi-automated algorithm based on colour contrast analysis, as described in Lai *et al*.^39^. Briefly, the algorithm tracked the trajectories of the preferred paw on the sagittal (x, y) plane by identifying the position of the paw previously painted with a green non-toxic dye (Stabilo Boss, Stabilo, Germany). To ensure consistency, only trajectories from successful trials (i.e. correct movements) were considered. Changes in the trajectories were quantified by the length of the whole trajectory (ArcLen) and the number of peaks in the tangential velocity profile (Smoothness). A detailed description of kinematic parameters is provided in Lai *et al*.^39^.

#### Open field test

Thirty minutes after DMCM treatment, animal were placed in a Plexiglass 60 cm × 60 cm arena with a white background and black walls for 10 minutes. The position of the animal during the test was automatically tracked using EthoVision XT (Noldus, Netherlands) and the total distance travelled was computed at the end of each experimental session.

### Intracortical Microstimulation (ICMS)

Animals were anesthetized using a ketamine (100 mg/kg) and xylazine (10 mg/kg) cocktail. The cortex was stimulated through a tungsten microelectrode (1 MΩ, FHC, USA), following a grid with nodes spaced 250 μm. At each penetration site, a 40 ms train of 13 cathodic current pulses (0.2 ms duty cycle) was delivered at 350 Hz from an electrically isolated, constant current stimulator (World Precision Instruments Inc., USA). The amplitude of the pulses was increased from a minimum of 20 μA to a maximum of 60 μA (with steps of 10 μA). Movements of several body parts were collected by a second experimenter, blinded to the stimulation coordinates in the grid.

#### Data Analysis

Data collected during ICMS were analyzed through a custom made algorithm developed in Matlab (Mathworks, USA) in order to obtain body part maps, multiple-site analysis and Transition Index (see supplementary information).

### Immunohistochemical analysis

At the end of the experiment, all animals were transcardially perfused with 4% paraformaldehyde. Brains were cut using a sliding microtome (Leica, Germany) to obtain 50-μm thick coronal sections that were used for immunostaining (see supplementary information). The number of Perineuronal Nets, Parvalbumin- and Somatostatin-positive neurons was analysed using a fluorescence microscope (Zeiss, Germany) with a 10x objective, counting in a 200 μm wide cortical column drawn at the medial and lateral edge of the ischemic tissue (Fig. 5) by Stereo Investigator software (MBF Bioscience, USA). Three sections per animal were analysed. The V-GAT e V-GluT1 signals were acquired using a confocal microscope (Leica, Germany) with a 63x objective and a 2.5 digital zoom, in medial-superficial/deep and lateral-superficial/deep regions in the perilesional tissue (Fig. 5). Three fields (95 μm × 95 μm) for each location were acquired (three sections per animal). Acquired images were processed using Metamorph software (Molecular devices, USA)to analyze the mean fluorescence in the entire image and in puncta rings regions by an experimenter blinded to the group^18^,^85^,^86^. To minimize variations due to the different quality of the immunostaining in individual mice/sections, fluorescence in perilesional areas was normalized to values calculated in three reference fields taken in the basal cortices of each analyzed coronal section.

To quantify the lesion volume, 1 out of every 6 sections was stained with Hoechst 33258 (Sigma-Aldrich, USA). The ischemic region was contoured using a 10x objective and its area measured. The lesion volume for each animal was calculated by summing up all damage areas and multiplying the number by section thickness and by 6 (the spacing factor). A total infarction volume in mm^3^ is given as the mean ± standard error of all analyzed animals.

### DMCM treatment

DMCM (methyl-6,7-dimethoxy-4-ethyl-beta-carboline-3-carboxylate) (Tocris, United Kingdom) was reconstituted in sterile water to obtain a concentration of 4 mg/ml. The DMCM treatment started at day 3 post lesion and continued up to day 8. DMCM (1.5 mg/kg) was delivered intraperitoneally under brief isoflurane anaesthesia twice a day, 10 hours apart. Behavioural effects of this dose of DMCM were verified in naïve mice by quantifying total distance travelled in the open field.

### Statistical Analysis

All statistical tests were performed using SigmaPlot 11.0 (Systat Software Inc, USA). For behavioural tests (gridwalk test, Schallert cylinder test, open field and skilled reaching test) a Two Way Repeated Measures ANOVA was used followed by a Tukey’s Test. For Immunohistochemical analysis One Way ANOVA was used followed by a Holm-Sidak test, or ANOVA on ranks followed by Dunn’s test when the sample failed the normality test. For intracortical microstimulation data a T-test was used. All statistical analyses were performed on raw data (alpha value 0.05).

## Additional Information

**How to cite this article**: Alia, C. *et al*. Reducing GABA_A_-mediated inhibition improves forelimb motor function after focal cortical stroke in mice. *Sci. Rep.* **6**, 37823; doi: 10.1038/srep37823 (2016).

**Publisher's note:** Springer Nature remains neutral with regard to jurisdictional claims in published maps and institutional affiliations.

## Supplementary Material

Supplementary Information
