# Supplementary material for: Reducing GABAA-mediated inhibition improves forelimb motor function after focal cortical stroke in mice
Source: Sci Rep. 2016 Nov 29;6:37823. doi: 10.1038/srep37823 (PMC5126677; doi:10.1038/srep37823)
Supplement: Supplementary Information [file srep37823-s1.pdf]

Reducing $GABA_A$-mediated inhibition improves forelimb motor function after focal cortical stroke in mice

Claudia Alia[1,2], Cristina Spalletti[2], Stefano Lai[3], Alessandro Panarese[3], Silvestro Micera[3,4], Matteo Caleo[2]*

[1]Scuola Normale Superiore, 56126, Pisa, Italy

[2]CNR Neuroscience Institute, 56124, Pisa, Italy

[3]Scuola Superiore Sant'Anna, Translational Neural Engineering Area, The BioRobotics Institute, 56025, Pontedera (PI), Italy

[4]Ecole Polytechnique Federale de Lausanne (EPFL), Bertarelli Foundation Chair in Translational NeuroEngineering Laboratory, Center for Neuroprosthetics and Institute of Bioengineering, CH-1015 Lausanne, Switzerland

Supplementary information

Photothrombotic lesion

The photothrombotic lesion was induced as previously described[1]. Briefly, animals were anesthetized with Avertin (20ml/kg, 2,2,2 tribromoethanol 1.25%; Sigma-Aldrich, USA) and placed in a stereotaxic apparatus. After a midline scalp incision, the bone was carefully dried and cleaned. Rose Bengal (0.2 ml of a 10mg/ml solution in PBS; Sigma Aldrich) was injected intraperitoneally. After 5 min, the brain was illuminated through the intact skull for 15 min using a cold light source (ZEISS CL 6000) linked to a 20X objective that was positioned 0.5 mm anterior and 1.75 mm lateral from Bregma (i.e. in correspondence with the caudal forelimb area; Tennant et al. 2011; Vallone et al. 2016). Sham animals underwent scalp incision and Rose Bengal injection but no light irradiation. At the end of the surgery, the skin was sutured and mice were allowed to awaken from anaesthesia.

Intracortical Microstimulation (ICMS)

Animals were anesthetized using a ketamine (100 mg/kg) and xylazine (10mg/kg) cocktail. A stable level of anaesthesia was maintained delivering 1/10 of the starting dose every 30 minutes. Animals were placed in a stereotaxic apparatus, the skull was exposed and a craniotomy (extending 3 mm and 4mm in the medio-lateral and antero-posterior direction, respectively) was performed in the ipsilesional hemisphere.

The cortex was stimulated through a tungsten microelectrode (1 MΩ, FHC, USA), inserted slowly into the brain at 700 µm depth for each stimulation point, following a grid with nodes spaced 250 µm. The ground electrode was placed under the skin of the neck. As reported in Tennant et al., 2011, at each penetration site, a 40 ms train of 13 cathodic current pulses (0.2 ms duty cycle) was delivered at 350 Hz from an electrically isolated, constant current stimulator (World Precision Instruments Inc., USA) guided by an electronic board (National Instruments Corp, USA). The amplitude of the pulses was increased from a minimum of 20 µA to a maximum of 60 µA (with steps of 10 µA). Movements of several body parts were collected by a second experimenter,

blinded to the stimulation coordinates in the grid. At the end of the ICMS procedure, the animal was sacrificed and the brain dissected for histology.

Data Analysis

Data collected during ICMS were analyzed through a custom made algorithm developed in Matlab (Mathworks, USA).

The body part (BP) maps (e.g. the stimulation maps for contralateral forelimb, contralateral hindlimb and tail) were obtained as follows. For each animal a matrix, with the same size of the stimulation grid (240 elements), was created for each BP and different stimulation current. Every element of the matrix indicates the presence/absence of the movement referred to the specific BP. The matrices of the single animals were then averaged into a mean map of 240 squares, each one depicted with a colorimetric index showing the probability to evoke the BP movement (Probability of Activation – *PA*). For each *j-th* site (j=1,..,NS, where NS is the number of stimulated sites) the *PA* was computed as follows:

$$PA_j = \frac{Ra_j}{Na}$$

where Ra_j is the number of animals showing the BP movement following the stimulation of the *j-th* site and *Na*is the total number of animals for each experimental group.

To improve the visual resolution, the matrix was 10-fold upsampled and Gaussian filtered. Contralateral forelimb (FL), contralateral hindlimb (HL) and tail (TL) showed a high and widespread activation after stroke (see Results). Thus we merged their BP maps to indentify the common activation areas before and after lesion. To study the maximum of the activation, the three BP maps computed with a 60 μA current amplitude were used. The maps were then thresholded in PA by considering a 50% threshold value, and finally overlapped. A colorimetric index was used to classify all of the possible cases observed (FL, FL+HL, FL+Tail, TL, HL+TL, FL+HL+TL). To smooth such maps, borders were manually drawn on the basis of matlab computed maps.

Since the distance between adjacent sites was 250 μm, the areas of 250 x 250 μm^2 centred at the each site were considered to calculate the *Percentage of Responding Area* (i.e. percentage of

stimulated area which elicits the BP movement, for a specific amplitude of stimulation current) of the whole maps:

$$\% \; of \; Responding \; Area = \frac{K \times (250 \; \mu m)^2}{Total \; Area} \times 100$$

where *K*is the number of responsive sites and *Total Area* is defined as $NS \times (250 \; \mu m)^2$, where *NS* is number of total stimulated sites.

For each animal, the *Minimum Current Threshold* was defined as the mean lowest current amplitude needed to evoke a movement of a specific BP.

Multiple sites analysis was performed taking into consideration 3 BPs: the contralateral forelimb, the contralateral hindlimb and the tail.. For each stimulation intensity, the analysis was performed by clustering sites into 3 classes: (i) sites evoking movements of a single BP (i.e. only forelimb); sites evoking movements of two (ii) or three (iii) BPs at the same time (i.e. forelimb, hindlimb and tail).

We also quantified how each cortical site changes its forelimb preference after the ischemic injury. We were interested in those sites that maximized the difference in forelimb activation probability in sham and stroke animals, i.e. those sites that had a high (or low) probability to evoke a forelimb movement in the sham group and a low (or high) probability to elicit forelimb movement after stroke. Thus, we defined a novel parameter, the Transition Index (TI), which shows the sham-stroke difference in forelimb activation probability. In order to quantify the TI amplitude, which shows the extent of the sham-stroke change, the average probability matrices of forelimb activation (*fPA*) and non-activation (*nfPA*) in sham and stroke groups were computed with a stimulation current of 30 µA. The *fPA* matrix is the PA matrix (see previous definition) computed with BP = {forelimb}, whereas *nfPA* is the *PA* matrix with BP = {non-forelimb}. For each *j-th* site (j=1,..,N, where N=240*)*, the amplitude of the TI was defined as an Euclidean distance:

$$TI_j = \sqrt{\left(\Delta P_{(sham)\,j}\right)^2 + \left(\Delta P_{(stroke)\,j}\right)^2}$$

where $\Delta P_{(sham)\,j} = (fPA_j - nfPA_j)$ for sham animals, whereas $\Delta P_{(stroke)\,j} = (fPA_j - nfPA_j)$ for stroke animals. Accordingly to this definition the TI value is defined within a range of $\pm\sqrt{2}$. Since we focused only on sites that inverted their ΔP after stroke (i.e. $\Delta P_{(sham)}$ and $\Delta P_{(stroke)}$have different signs),we did not consider cases with $\Delta P_{(sham)}$ and $\Delta P_{(stroke)}$with the same sign, thus arbitrarily setting the TI of these sites to zero. In the other cases, the sign of TI (sTI) that shows the direction of the change, was assigned accordingly as follow: (i) when $\Delta P_{(sham)}$ >0 and $\Delta P_{(stroke)}$<0 then sTI was defined positive (i.e. loss of forelimb movements); (ii) when $\Delta P_{(sham)}$ <0 and $\Delta P_{(stroke)}$ >0 then sTI was defined negative (i.e. gain of forelimb).

Antibodies for immunohistochemistry

In the paper we used following antibodies and concentrations: NeuN (1:1,000, Millipore, Germany), Perineuronal Nets (Wisteria floribunda agglutinin, WFA; 1:100, Sigma, USA), Parvalbumin (1:300, Synaptic Systems, Germany), Somatostatin (1:400, Millipore, Germany), V-GAT (1:800, Synaptic Systems, Germany), V-GluT1 (1:1,000, Synaptic Systems, Germany).

References

1. Lai, S. *et al.* Quantitative Kinematic Characterization of Reaching Impairments in Mice After a Stroke. *Neurorehabil. Neural Repair* **29,** 382–392 (2015).
2. Tennant, K. A. *et al.* The organization of the forelimb representation of the C57BL/6 mouse motor cortex as defined by intracortical microstimulation and cytoarchitecture. *Cereb. Cortex* **21,** 865–876 (2011).
3. Vallone, F. *et al.* Post-Stroke Longitudinal Alterations of Inter-Hemispheric Correlation and Hemispheric Dominance in Mouse Pre-Motor Cortex. *PLoS One* **11,** e0146858 (2016).

Supplementary Figures

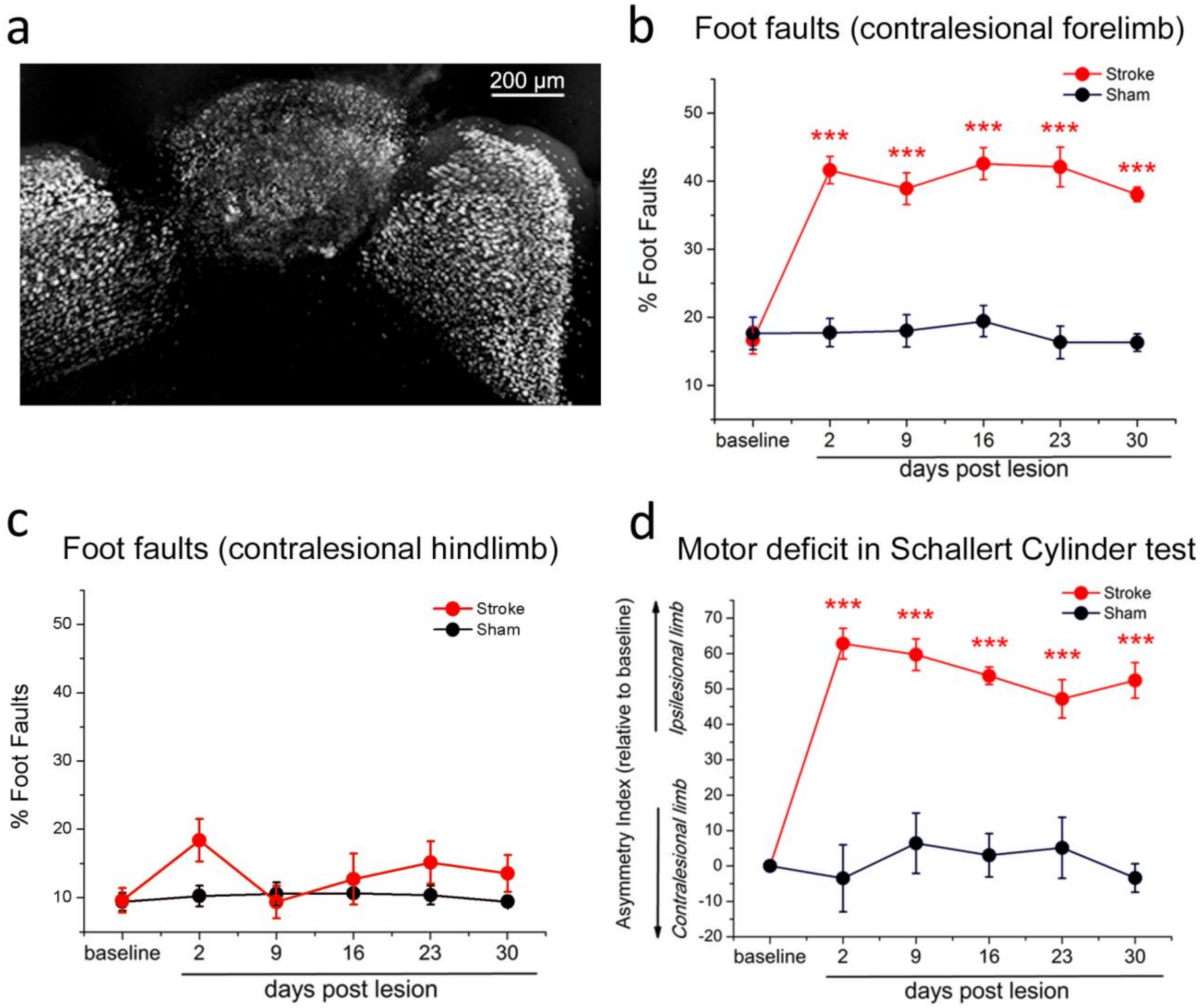

Fig. S1. Stroke-induced neuronal damage and associated motor deficits. (**a**) Representative NeuN immunostaining of the phothrombotic lesion in a coronal brain section. The boundaries of the ischemic region are clearly evident and the core of the lesion shows loss of NeuN staining Scale bar = 200 µm. (**b**, **c, d**) Pre- and post-lesion performance of the stroke (red, n=11) and sham (black, n=9) groups measured as the percentage of contralesional forelimb (**b**) and hindlimb (**c**) foot faults in the gridwalk task and the Asymmetry Index in the Schallert cylinder test (**d**). Note persistent motor deficits in the stroke mice. The sham animals maintained a low proportion of foot faults and a balanced use of their forelimbs throughout the testing period. Data are mean ± SE. Two Way RM ANOVA followed by Tukey test, $p<0.001$ with respect to baseline.

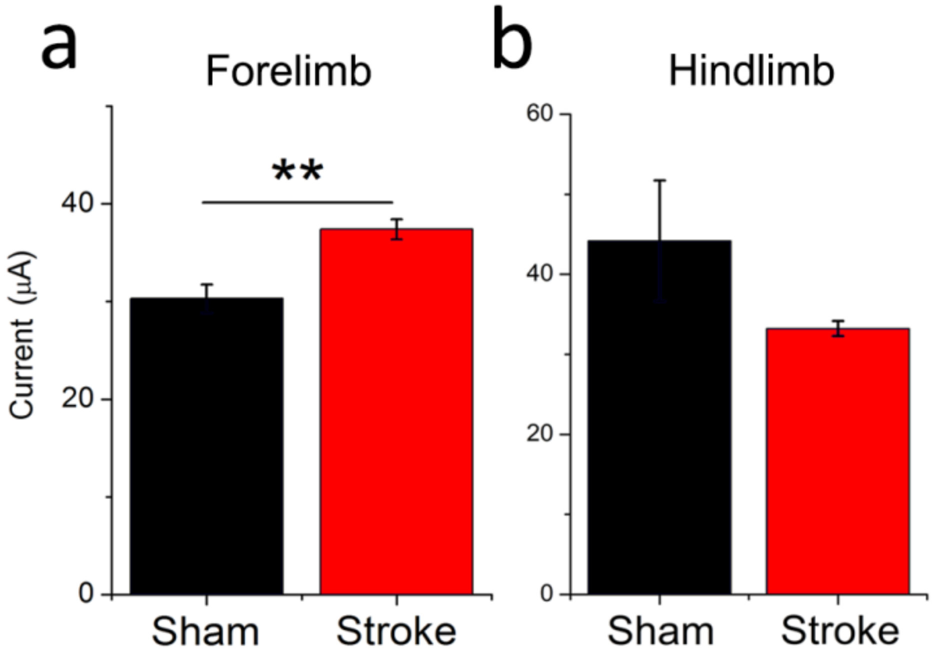

Fig. S2. Stroke increases the threshold current required to evoke forelimb movements. Minimum current amplitude required to elicit movement of the contralateral forelimb (**A**) or hindlimb (**B**) in the sham (n=9) and stroke group (n=6). The current threshold of the residual forelimb sites in perilesional areas were higher than normal (t-test, $p<0.01$). A tendency for reduction was detected for the current intensities required to evoke hindlimb movements, but this trend only approached statistical significance (t-test, $p=0.148$). Data are mean ± SE.

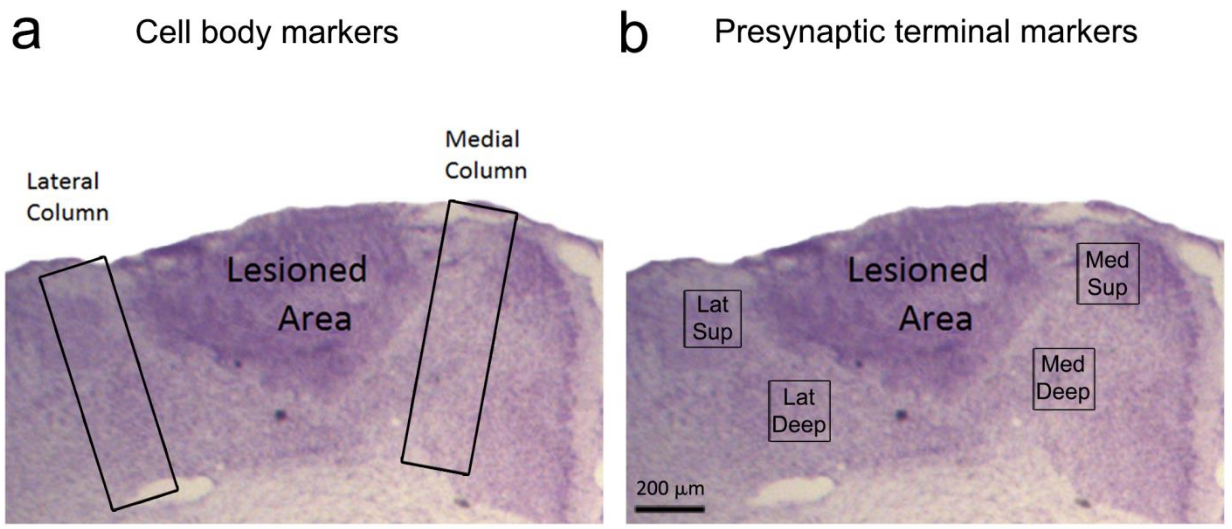

Fig. S3. Location of counting boxes for the immunohistochemical analyses. (**A**) Cell-counting scheme for PNNs, PV-+ and SOM-+ cells superimposed on a representative Nissl staining of a cortical section. Two cortical columns (200µm wide) adjacent to the lesion have been analyzed for each section. (**B**) Scheme for the fluorescence analysis of the presynaptic terminal markers. Four regions has been taken into consideration: Lateral-superficial layers (Lat Sup), Lateral-deep layers (Lat Deep), Medial-superficial layers (Med Sup) and Medial-deep layers (Med Deep).

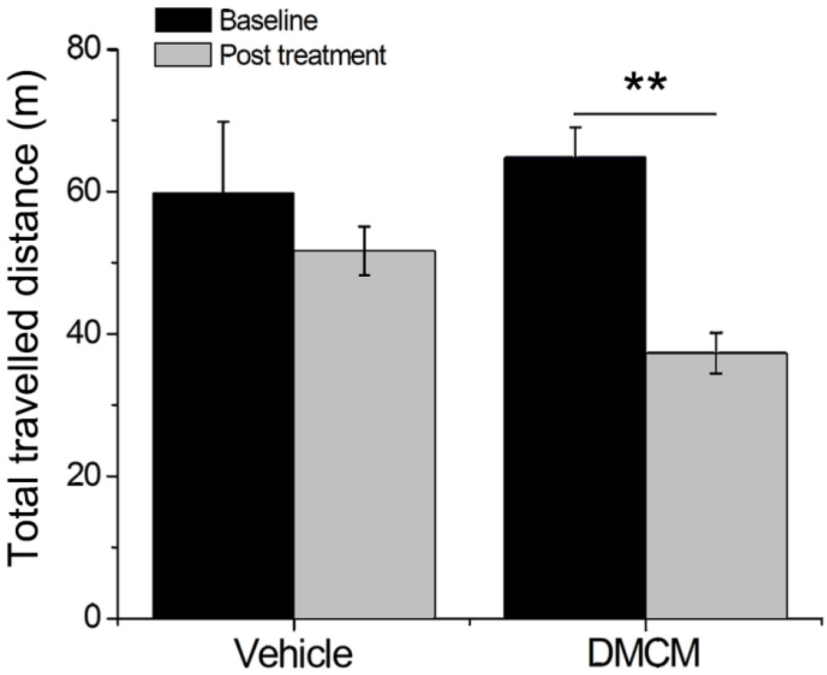

Fig. S4. Effect of DMCM on exploratory activity in the open field. Total distance travelled by healthy animals treated with either DMCM (1.5 mg/kg) (n=3) or vehicle (n=3). Measurements were taken before (black) and after treatment (grey). DMCM produces a very significant reduction of locomotor activity (two way RM ANOVA, followed by Holm-Sidak test, $p<0.01$) while vehicle had no significant impact ($p=0.175$).

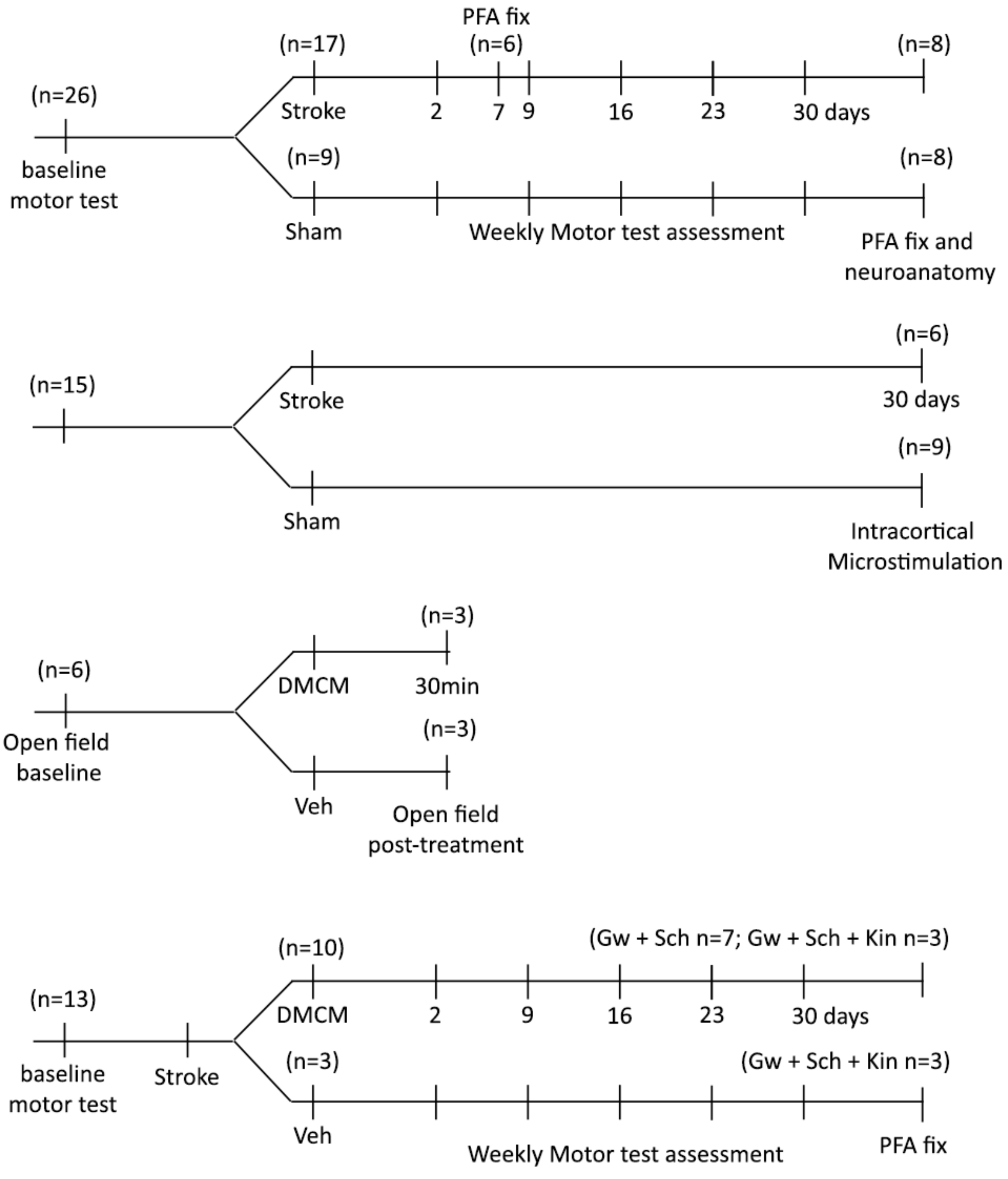

Fig. S5. Experimental Design. Animals were randomly subdivided in groups for studying stroke-induced motor deficits, anatomical changes and motor map reorganization. A subset of animals was used to evaluate DMCM effects on healthy animals and finally a group was used to evaluate the DMCM effect on the post-stroke motor recovery. PFA, paraformaldehyde; GW, Gridwalk test; Sch, Schallert Cylinder test; Kin: skilled reaching test and kinematic analysis; Veh, vehicle.
